# An update of the macaque testis proteome

**DOI:** 10.1016/j.dib.2015.08.029

**Published:** 2015-09-04

**Authors:** Tao Zhou, Yueshuai Guo, Zuomin Zhou, Xuejiang Guo, Jiahao Sha

**Affiliations:** State Key Laboratory of Reproductive Medicine, Nanjing Medical University, Nanjing 210029, PR China

**Keywords:** Reanalysis, Rhesus macaque, Tandem mass spectrometry, Testis, Y chromosome

## Abstract

The genome sequence of rhesus macaque is a draft version with many errors and is lack of Y chromosome annotation. In the present dataset, we reanalyzed the previously published macaque testis proteome. We searched for refined protein sequences, potential Y chromosome proteins and transcripts predicted proteins in addition to the latest Ensembl protein sequences of macaque. A total of 74,433 peptides corresponding to 9247 protein groups were identified, and the data are supplied in this paper. The updated version of macaque testis proteome provided evidences for predicted genes or transcripts at the peptide level. It can be used for further in-depth proteogenomic annotation of macaque genome and is useful for studying the mechanisms of macaque spermatogenesis.

Specifications table

**Table:** 

Subject area	Biology
More specific subject area	Reproductive biology
Type of data	Table
How data was acquired	Mass spectroscopy, LTQ Orbitrap Velos (ThermoFinnigan, San Jose, CA)
Data format	Analyzed
Experimental factors	Protein samples were reduced with 5 mM DTT, alkylated with 14 mM IAA, and digested using sequencing grade trypsin.
Experimental features	Digested peptides were separated using an ion exchange column prior to HPLC–MS/MS with subsequent data analysis of protein identification.
Data source location	Nanjing, China
Data accessibility	The data are with this paper.

Value of the data

• The macaque testis proteome was reanalyzed using refined and integrated protein sequences.
• A total of 8439 new peptides than previously reported were identified.
• Novel experimental evidences for the expression of macaque predicted genes and transcripts.

## 1 Data, experimental design, materials and methods

### 1.1 Experimental design

The rhesus macaque (*Macaca mulatta*) is a non-human primate model which is widely applied in biomedical researches including reproductive physiology and male contraception [1,2]. We previously constructed the proteome of adult macaque testis by liquid chromatography-tandem mass spectrometry (LC-MS/MS) [3]. However, the genome sequence of macaque is a draft version with many errors and is lack of Y chromosome annotation [4,5]. The protein sequence database for previous macaque testis proteome is released in November 2010 (Ensembl 60) [6]. Recently, a refined macaque genome assembly and the transcriptome of macaque testis were released [7]. Thus, it is necessary to update the proteome to obtain a more precise understanding of the protein composition of macaque testis. In the present dataset, we used the latest sequence databases to reanalyze the proteome of macaque testis.

## 2 Materials and methods

### 2.1 Testis samples and ethics statement

Testis samples were obtained from two adult male macaques with normal fertility via operation. All procedures were approved in advance by the Institutional Animal Care and Use Committee of Kunming Biomed International, and the animal facilities were accredited by the Association for Assessment and Accreditation of Laboratory Animal Care International.

### 2.2 Protein extraction and LC-MS/MS analysis

For protein extraction, decapsulated testes were cut into small pieces and dissolved in 8 M Urea, 75 mM NaCl, 50 mM Tris, 1 mM NaF, 1 mM β-glycerophosphate, 1 mM sodium orthovanadate, 10 mM sodium pyrophosphate, 1 mM PMF, and 1% (v/v) protease inhibitors cocktail. And then testis proteins were extracted via ultrasonic method. The extracted proteins from the two macaques were individually used for subsequent proteomic analyses.

Testis proteins were reduced with 5 mM DTT/25 mM NH_4_HCO_3_ at 56 °C for 20 min, alkylated with 14 mM IAA/25 mM NH_4_HCO_3_ in the dark at room temperature for 30 min. Then the protein mixture was diluted using 25 mM Tris–HCl (pH 8.2) to reduce the concentration of urea to 1.6 M, mixed with trypsin (sequencing grade modified trypsin, Promega, WI, USA) at a trypsin: protein ratio of 1:200, and was incubated at 37 °C overnight. TFA was added at a final concentration of 0.4% to terminate the digestion reaction. Extracts were subjected to C18 SPE on Sep-Pak cartridges. The resulting peptides were dissolved in SCX Buffer A.

The peptide mixture was separated using an ion exchange PolySulfoethyl A HPLC column (2.1 mm ID×20 cm, 5 μm, 200 Å, PolyLC) with the UltiMate® 3000 HPLC systems at a flow rate of 200 μl/min. Peptide analysis was performed using the LTQ Orbitrap Velos (Thermo Finnigan, San Jose, CA) coupled directly to an LC column. An MS survey scan was obtained for the m/z range 350–1800, and MS/MS spectra acquired from the survey scan for the 20 most intense ions (determined using Xcalibur mass spectrometer software in real time). Dynamic mass exclusion windows of 60 s were used, with siloxane (m/z 445.120025) as a lock mass.

### 2.3 Data analysis

The raw files were processed using MaxQuant version 1.3.0.5 [8]. The database search was performed against the combined sequences from the Ensembl macaque protein sequences (updated in May 2015) [6], the refined macaque protein sequences (MacaM_v7.6.8) [7], conceptual translated protein sequences using macaque transcriptome [7], potential Y chromosome expressed proteins extracted from the UniProt database (updated in June 2015) [9], and the standard MaxQuant contaminant sequences. Carbamidomethyl (C) was set as a fixed modification. Variable modifications were Oxidation (M) and N-term Acetylation (Protein N-term).The mass tolerance for precursor ions was set to 20 ppm at the first search as applied in Maxquant for initial mass recalibration. For the main search, the mass tolerance for precursor ions was set to 6 ppm. The mass tolerance for fragment ions was set to 0.5 Da. Enzyme specificity was set to be fully cleaved by trypsin, and the maximum missed cleavage sites permitted was two. The false discovery rate (FDR) of the identification was estimated by searching against the databases with the reversed amino acid sequences. The site, peptide and protein FDR were all set to 0.01. The minimum peptide length required was six. At least one unique peptide is required for protein report.

## 3 Data

**Fig. 1:**
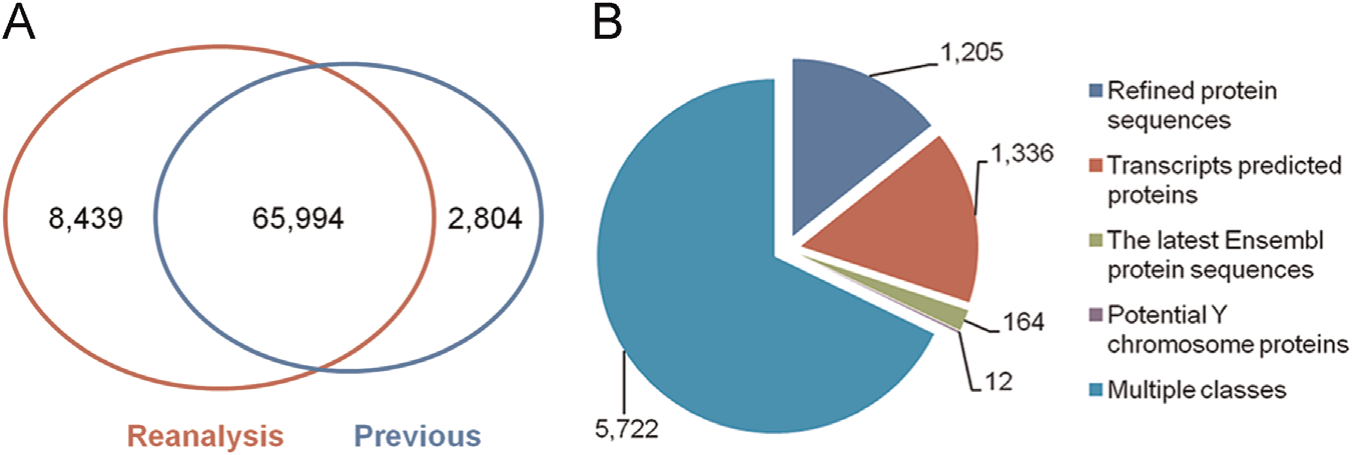
Summary of the updated macaque testis proteome. (A) Comparison of identified peptides between the present dataset with previous study. (B) Distribution of novel identified peptides in different sequence databases.

In summary, a total of 74,433 peptides corresponding to 9247 protein groups were identified as the new version of macaque testis proteome based on the results of two biological replicates. The detailed information including protein accessions, peptide counts, sequence coverage, score, and intensity for each identified protein group is listed in Supplementary Table 1. The detailed information including protein accessions, peptide sequence, charges, score and intensity for each peptide is listed in Supplementary Table 2. Compared with previous study, the present dataset identified 8439 more peptides (Fig. 1A). Most of the novel peptides could be used to refine current macaque gene annotation (Fig. 1B). The updated macaque testis proteome provided evidences for predicted genes or transcripts at the peptide level. It can be used for further in-depth proteogenomic refinement of macaque genome and also provides resources for studying the mechanisms of macaque spermatogenesis.

## Funding sources

This work was supported by Grants from the 973 Program (2012CBA01306), and the Chinese Natural Science Funds (81222006).
